# Contact Lens Health Week — November 17–21, 2014

**Published:** 2014-11-14

November 17–21, 2014, marks the first annual Contact Lens Health Week. In close collaboration with partners from clinical, public health, industry, and regulatory sectors, CDC has developed a campaign to promote healthy contact lens wear and care practices that can help reduce the risk for eye infections and complications associated with poor contact lens hygiene.

Microbial keratitis is a serious, sometimes blinding, eye infection often associated with poor contact lens hygiene. CDC has published a report on the first estimate of the burden of keratitis, including microbial keratitis, and contact lens disorders in the United States, using data from national outpatient and emergency department databases (1). The report finds that episodes of keratitis and contact lens disorders result in an estimated 930,000 outpatient visits and 58,000 emergency department visits annually that cost $175 million in direct health care expenditures.

Established, modifiable risk factors for microbial keratitis, such as overnight contact lens wear, poor contact lens storage case hygiene, and infrequent storage case replacement (2,3), indicate that this serious and costly eye infection is largely preventable. As such, patient education about healthy contact lens wear and care practices is essential and warranted. Additional information on Contact Lens Health Week and the proper wear and care of contact lenses is available at http://www.cdc.gov/contactlenses.
